# Suppression of the Nuclear Factor Eny2 Increases Insulin Secretion in Poorly Functioning INS-1E Insulinoma Cells

**DOI:** 10.1155/2012/460869

**Published:** 2012-05-10

**Authors:** P. Dames, M. Weise, R. Puff, B. Göke, K. G. Parhofer, J. Seissler, A. Lechner

**Affiliations:** ^1^Medizinische Klinik II, Ludwig-Maximilians-Universität, München 80336, Germany; ^2^Medizinische Klinik IV, Ludwig-Maximilians-Universität, München 80336, Germany

## Abstract

Eny2, the mammalian ortholog of yeast Sus1 and drosophila E(y)2, is a nuclear factor that participates in several steps of gene transcription and in mRNA export. We had previously found that Eny2 expression changes in mouse pancreatic islets during the metabolic adaptation to pregnancy. We therefore hypothesized that the protein contributes to the regulation of islet endocrine cell function and tested this hypothesis in rat INS-1E insulinoma cells. Overexpression of Eny2 had no effect but siRNA-mediated knockdown of Eny2 resulted in markedly increased glucose and exendin-4-induced insulin secretion from otherwise poorly glucose-responsive INS-1E cells. Insulin content, cellular viability, and the expression levels of several key components of glucose sensing remained unchanged; however glucose-dependent cellular metabolism was higher after Eny2 knockdown. Suppression of Eny2 enhanced the intracellular incretin signal downstream of cAMP. The use of specific cAMP analogues and pathway inhibitors primarily implicated the PKA and to a lesser extent the EPAC pathway. In summary, we identified a potential link between the nuclear protein Eny2 and insulin secretion. Suppression of Eny2 resulted in increased glucose and incretin-induced insulin release from a poorly glucose-responsive INS-1E subline. Whether these findings extend to other experimental conditions or to in vivo physiology needs to be determined in further studies.

## 1. Introduction

Diabetes mellitus Type 2 (T2D) is usually caused by peripheral insulin resistance together with inadequate insulin secretion by the pancreatic beta cells [1]. The latter represents the combination of a primary, probably genetically determined, failure to adapt to increased insulin requirements and secondary beta cell defects caused by, for example, glucotoxicity and amyloid depositions [2, 3]. Impaired insulin secretory function plays a major role both in diabetes manifestation and progression [4]. Two groups of antidiabetic drugs that directly target beta cell function are currently in clinical use. The sulfonylureas act via glucose-independent closure of K_ATP_ channels, and the glucagon-like peptide 1 (GLP-1) analogues and enhancers augment insulin secretion via the GLP-1 receptor and cAMP. Because of the importance of beta cell function for both the pathogenesis and the treatment of T2D, the molecular pathways regulating insulin secretion are of high interest.

Eny2 is a small (12 kD), highly conserved nuclear protein that is probably expressed ubiquitously. Its orthologs in yeast and drosophila are termed Sus1 and E(y)2, respectively. Eny2 is a component of the SAGA/TFTC histone-acetyltransferase complex that acts in transcriptional activation [5]. It is also a part of the TREX-2/AMEX complex that mediates mRNA export from the nucleus [6]. Additional functions include participation in mRNP formation [7] and the barrier activity of genomic insulators [8]. Eny2 is therefore thought to be a coupling factor that integrates mRNA transcription, processing, and nuclear export [9].

We identified Eny2 as a differentially expressed gene in microarray expression profiles of pancreatic islets from pregnant and control mice (A.L.; unpublished). Pregnancy is associated with fundamental changes of beta cell mass and function in the maternal rodent pancreas. We therefore hypothesized that Eny2 could have regulatory roles in islet endocrine cells. In the present study we tested this hypothesis in rat INS-1E insulinoma cells.

## 2. Materials and Methods

### 2.1. Islet Isolation

To confirm Eny2-expression in the islets of Langerhans, islets from C57Bl/6 mice (Charles River laboratories) were isolated by collagenase digestion of the pancreas and selected by hand picking under a stereoscope as previously described [10], followed by RNA extraction and rtPCR.

### 2.2. Cell Culture, Overexpression and Knockdown of Eny2

INS-1E cells (passages 78–92) [11] were a generous gift from Maechler and Wollheim (Geneva) and were cultured at 37°C and 5% CO_2_ in a humidified atmosphere in RPMI 1640 media (containing 11,1 mmol/l glucose (PAA)) supplemented with 5% heat-inactivated fetal calf serum (PAA), 100 IU/mL penicillin (PAA), 100 *μ*g/mL streptomycin (PAA), 10 mM HEPES, 1 mM sodium pyruvate (PAA), and 50 *μ*M *β*-mercaptoethanol (Sigma-Aldrich). Cells were passaged every 5–7 days by trypsinization.

Transient overexpression of mouse Eny2 was done with a pcDNA3 expression vector, which contained the mouse Eny2-ORF under the control of a CMV-promotor. Roti Fect Plus transfection reagent (Roth) was used following the manufacturer's protocol. In brief, INS-1 cells were seeded in 24-well culture plates at a density of 1.5 × 10^5^ cells/well and transfected within 1 h after seeding with a mixture of antibiotic-free culture media supplemented with transfection reagent and either control-vector (pcDNA3) or pcDNA3-Eny2/pcDNA3-Eny2-HA. The transfection media was replaced with normal culture media after 24 hours.

To knock down endogenous Eny2 protein, transient transfections with Eny2-specific siRNA (Silencer select; Ambion) was performed using Lipofectamin RNAi MAX (Invitrogen) following the manufacturer's reverse transfection protocol. INS-1E cells were seeded in 24-well culture plates at a density of 1.5 × 10^5^ cell/well. The wells contained complete culture media without antibiotics premixed with control or Eny2-specific siRNA/Lipofectamin complexes (100 nM siRNA, 1,5 *μ*L Lipofectamin). Cells were then cultured without any further media replacement until the assays were done. 72–96 hours effective mRNA suppression was confirmed by rtPCR. The siRNA sequences were as follows: GCGAUUAACCAAAAGUUAA (Eny2-siRNA1), AAAGGACUAGAACACGUUA (Eny2-siRNA3), AAUUGUUCGACUUUCUUGG (control).

### 2.3. RNA Isolation and Semiquantitative rtPCR

Total RNA was isolated by RNeasy Mini Kit in combination with DNA removal columns obtained from Qiagen. cDNA was synthsized from total RNA with oligo(dT) primers and the ImProm-II Reverse Transcription system (Promega). Semiquantitative rtPCR was done with the Tag PCR Core Kit (Qiagen) and GAPDH, cyclophilin, and beta actin for normalization. Negative controls (-rt) were done for all reactions and PCR products were sequenced to confirm their correct identity. The minimal necessary cycle number was determined for each amplicon to achieve a reliable semiquantitative amplification. The primer sequences were  CTGTTGATGACTTGGTGGC and TTCAGCCACCAAGTCATCAA-3′ for Eny2 (mouse), CTTAGGTGCCCGAGCTACTG and TTGCTAACCACCATCACCGC for Eny2 (rat), AACTTTGGCATTGTGGAAGG and ACACATTGGGGGTAGGAACA for GAPDH (mouse), GGCATTGCTCTCAATGACA and TGTGAGGGAGATGCTCAGT for GAPDH (rat), CACCTTTGTGGTCCTCACC and CCAGTTGGTAGAGGGAGCA for insulin 1 (rat), AATTTCATCATCGCCCTCTG and GTCTCTGATGACCCCAGGAA for Glut2 (rat), TCAGGACTTGCACTTTCACG and ACTCTCCTGGGGTCTGGTCT for glucokinase (rat), CCTGGAGGCCATTGTAGAAA and GGACATTGTTGCTGATGGTG for phosphofructokinas (rat), GTGTGTGGTCTTTGGGAAGG and TACAGGGTATTGCGAGCAGA for cyclophilin (rat), AGCCATGTACGTAGCCATCC, and GCTGTGGTGGTGAAGCTGTA for *β*-actin (rat).

### 2.4. Measurement of Insulin Secretion and Insulin Content

Following a transfection period (72 h for overexpression experiments; 72–96 h for siRNA-mediated knockdown) INS-1E cells were washed twice in Krebs-Ringer-Bicarbonate-Hepes buffer (KRBH; 135 mM NaCl, 3.6 mM KCl, 1.5 mM CaCl_2_, 0.5 mM NaH_2_PO_4_, 0.5 mM MgCl_2_, and 5 mM NaHCO_3_, 10 mM HEPES) buffer containing 0.1% BSA and were preincubated at 37°C for 30 min with the same buffer. Subsequently, cells were incubated for another 30 min in 0,5 mL KRBH buffer supplemented with 0.1% BSA, different glucose concentrations, and other secretagogues, as indicated in the figure legends (tolbutamide (Sigma); exendin-4 (Byetta, Lilly); Forskolin (Sigma), 8-(4-chlorophenylthio)-2′-O-methyladenosine-3′,5′-cyclic monophosphate and N6-benzyladenosine-3′,5′-cyclic monophosphate (Biolog Life Science), H-89 (Calbiochem)). Supernatants were collected for measurements of secreted insulin. The attached cells were extracted with lyses buffer (50 mM HEPES, 0.1% Triton X-100, 1 mM DTT, 1x protease inhibitor cocktail (Roche)) to determine the protein and insulin content. Insulin was assayed by ELISA (Millipore; Linco) following the manufacturer's protocol.

### 2.5. Glucose-Dependent MTT-Assay

Glucose metabolism was assessed by an MTT (C,N-diphenyl-N′-4,5-dimethyl thiazol 2 yl tetrazolium bromide) assay, which measures cytoplasmatic reduction equivalents resulting from glycolysis and mitochondrial oxidation [12]. 

INS-1E cells were reverse-transfected with either scrambled or targeting siRNA as described and seeded in 24-well culture plates. 72–96 h after transfection, cells were washed and preincubated with glucose-free KRBH buffer at 37°C for 30 min. Subsequently, cells were washed again and incubated for another 30 min period at 37°C with 0,5 mL KRBH buffer supplemented with different glucose concentrations and MTT reagent (0.5 mg/mL). For solubilization of the produced formazan crystals, the KRBH buffer was replaced by 1 mL isopropanol/10%-DMSO solution. The absorbance of the formazan was then measured with a photometer (Eppendorf) at 550 nm. 

### 2.6. Assessment of Cellular Proliferation/Viability

After an incubation time of 72 h, siRNA transfected INS-1E cells, seeded in 24-well cultures plates were incubated with MTT-reagent (0,5 mg/mL) without any further media replacement. After an incubation period of 1,5 h the media MTT solution was replaced by isopropanol/10% DMSO and 5minutes later the absorbance of produced formazan was measured at 550 nm.

### 2.7. cAMP Assay

For measurement of cAMP formation, INS-1E cells were treated with siRNA, seeded in 24-well plates and cultured for 72–96 h. Cells were then washed twice with KRBH buffer, preincubated with the same buffer at 37°C for 30 min, washed once again followed by incubation with 0.5 mL KRBH buffer containing 16.7 mM glucose with or without different concentrations of exendin-4 and 100 *μ*M IBMX (3-isobutyl-1-methylxanthine (Sigma)). cAMP was extracted after 5–15 minutes by adding 0.1 mol/l HCl/0.5% Triton X-100 to the cells. After a centrifugal step at 600 g for 10 min, the level of cAMP was measured in the supernatant by using a competitive cAMP ELISA (Thermo Scientific).

### 2.8. Statistical Analysis

All results are expressed as mean ± standard deviation. The unpaired *t*-test was used for comparisons of 2 experimental conditions. For 3 or more conditions one-way ANOVA followed by posttests of selected comparisons (Bonferroni method) was used.

## 3. Results

### 3.1. Eny2 mRNA Is Expressed in Mouse Pancreatic Islets and Rat Insulinoma Cells: Suppression by siRNA

Our experiments started from a microarray expression profiling experiment that compared C57BL/6 islets from day 12.5 pregnant and control females (A.L.; unpublished). ENY2 was significantly upregulated on day 12.5 when beta cell proliferation is at its highest level during the adaptation of the maternal pancreas to pregnancy.

**Figure 1:**
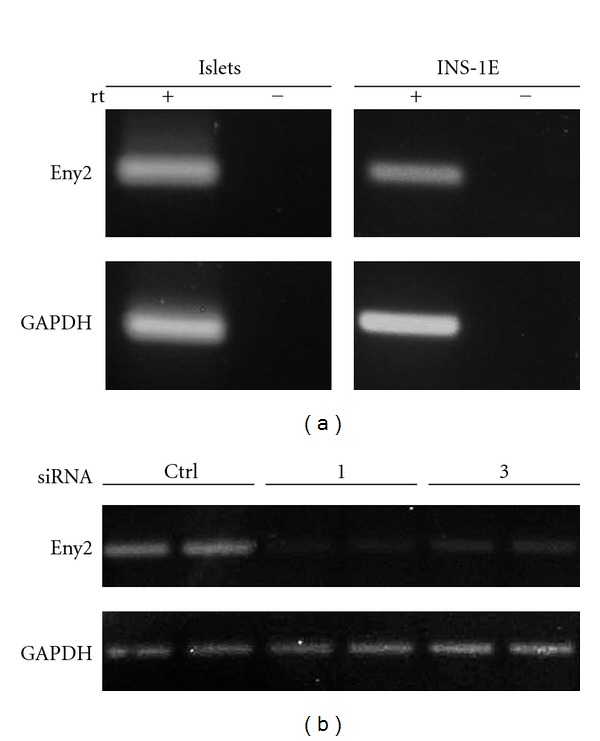
RT-PCR detection of Eny2 mRNA expression in mouse pancreatic islets and INS-1E insulinoma cells: knockdown by two specific siRNAs. (a) The mRNA of Eny2 was detected in isolated mouse pancreatic islets (left panel) and in INS-1E insulinoma cells (right panel). (b) Eny2 mRNA is efficiently knocked down 72 hours after transfection of INS-1E cells with 2 specific siRNAs (numbers 1 and 3) but not with a scrambled control siRNA.

We next confirmed the expression of Eny2 mRNA in islets from C57Bl/6 mice using rtPCR and also in rat INS-1E insulinoma cells [11] (Figure 1(a)). INS-1E cells were then used in functional studies. We first tested several Eny2-specific siRNAs for efficient gene knockdown in this cell line. Because no antibody towards mammalian Eny2 was available, we examined Eny2 expression on the mRNA level. siRNAs 1 and 3 suppressed Eny2 mRNA after 72–96 hours (Figure 1(b)) and were used in further experiments.

### 3.2. Eny2 Knockdown Results in Increased Glucose-Stimulated Insulin Secretion (GSIS) from Otherwise Poorly Functioning INS-1E Cells

**Figure 2:**
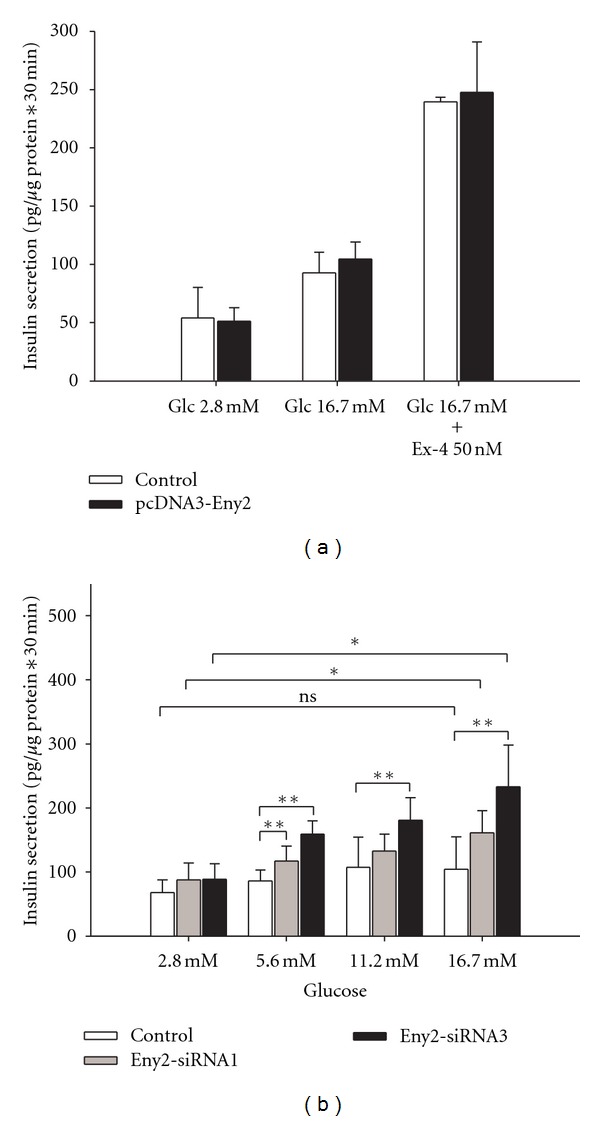
GSIS by INS-1E cells after overexpression of Eny2 and siRNA-mediated Eny2 knockdown. (a) Mouse Eny2 was transiently overexpressed in INS-1E cells by plasmid transfection. No change of glucose and exendin-4-induced insulin secretion was observed 72 hours after transfection (*n* = 3). (b) Glucose-dependent insulin secretion was increased 72–96 hours after transfection with two different Eny2-specific siRNAs compared to a control siRNA (*n* = 5). *P < 0.05, **P < 0.01, ***P < 0.001; the unpaired *t*-test was used for comparisons between two experimental conditions, one-way ANOVA with posttests when 3 or more conditions were analyzed.

To test if Eny2 affects the function of insulin-producing cells we first constructed an expression vector for mouse Eny2. (The protein sequences of mouse and rat Eny2 are identical.) No change in glucose and exendin-4-induced insulin secretion was observed after transient overexpression of mouse Eny2 (Figure 2(a)). During the course of these experiments we found however that the INS-1E cells in our lab had developed a low-glucose responsiveness compared to the original INS-1E line 1.7- versus 4.3-fold [11, Figure 2(a)]. We nevertheless also performed knockdowns of Eny2 in these cells using the two tested and effective gene-specific siRNAs. We compared glucose-stimulated insulin secretion in the knockdown cells to that of cells treated with a scrambled control siRNA. Insulin secretion was augmented with both Eny2-specific siRNAs in a glucose-dependent manner (Figure 2(b)). Stimulation of insulin secretion from 2.8 mM to 16.7 mM glucose was 1.9-fold for siRNA 1 (from 87 ± 26 pg/*μ*g protein∗30 min to 162 ± 34 pg/*μ*g protein∗30 min; *P* < 0,05) and 2.7-fold for siRNA 3 (from 88 ± 24 pg/*μ*g protein∗30 min to 233 ± 65 pg/*μ*g protein∗30 min; *P* < 0,05).

### 3.3. Insulin Content, Cellular Proliferation, and Key Components of Glucose Sensing Are Unchanged after Eny2 Suppression

**Figure 3:**
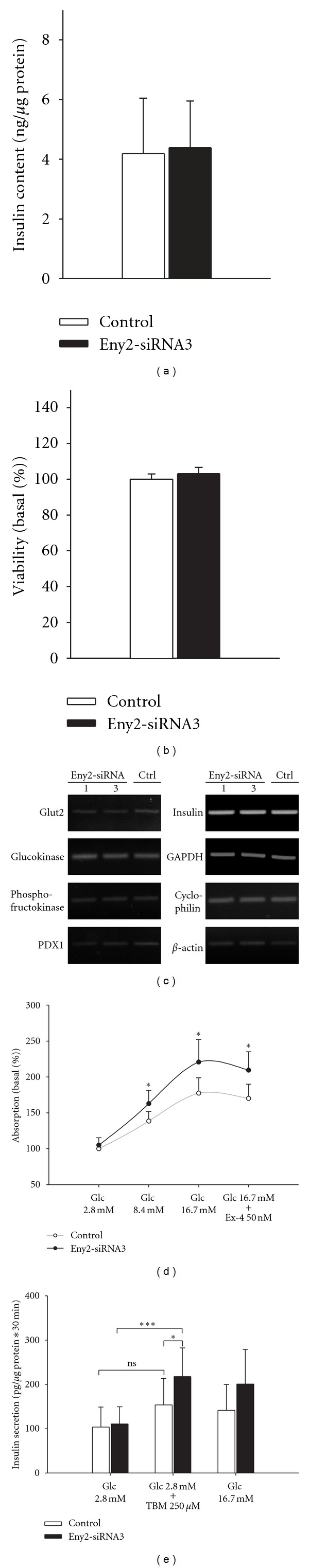
Insulin content, cellular proliferation, gene expression, glucose metabolism, and response to tolbutamide after Eny2 knockdown. (a) Cellular insulin content was measured 72 hours after transfection with control and Eny2-specific siRNA, respectively. Insulin content was comparable after both treatments (*n* = 3). (b) INS-1E cells were seeded at low density and in equal numbers per well and were transfected with either control or Eny2-specific siRNA. After 3 days cellular viability was quantified using MTT. No difference between the two treatments was observed indicating unchanged cellular proliferation (*n* = 3). (c) The expression of several key components of beta cell glucose sensing as well as of the transcription factor pdx1 and insulin itself were measured by semiquantitative rtPCR. GAPDH, cyclophilin, and beta actin were used for normalization. No difference in the expression level of any of the genes was seen. (d) Glucose-dependent cellular metabolism was examined by an MTT assay (*n* = 3). The increase of metabolism with rising glucose concentrations was augmented after Eny2 knockdown. (e) We also tested insulin secretion in response to the sulfonylurea secretagogue tolbutamide. Tolbutamide had a minimal effect in control cells but clearly augmented insulin secretion after Eny2 suppression. *P < 0.05, ***P < 0.001 unpaired *t*-test.

To examine the mechanism of increased GSIS after Eny2 knockdown we first measured cellular insulin content, which was unchanged (Figure 3(a)). Cellular proliferation was also not affected by Eny2 suppression. 72 hours after seeding equal numbers of INS-1E cells per well and transfection with either Eny2-specific siRNA or a control, cellular viability was comparable (Figure 3(b)). Cellular protein content was equally unchanged (data not shown). We next examined the expression levels of insulin, the transcription factor pdx1, and of several key components of glucose sensing (Glut2, glucokinase, phosphofructokinase) using semiquantitative rtPCR. GAPDH, cyclophilin and beta-actin were used as controls. No change in the expression levels of any of the genes was observed (Figure 3(c)).

### 3.4. Eny2 Knockdown Results in a Higher Glucose-Dependent Metabolic Activity

GSIS from pancreatic beta cells is coupled to glucose metabolism [13]. Therefore we wanted to test whether Eny2 knockdown affected the cells' metabolic activity. Glycolysis results in pyruvate production, which enters the mitochondria and is further metabolized in the tricarboxylic acid (TCA) cycle. This results in the production of the reducing equivalents NADH and FADH_2_ [13]. We used an MTT assay to measure glucose-dependent production of reducing equivalents and thus to indirectly quantify cellular glucose metabolism [12, 14]. We found that reduction of MTT increased with increasing glucose concentrations (2,8 to 16,7 mmol/l) in both the control and the Eny2-knockdown cell, but that the degree of increase was significantly higher with Eny2 suppression (Figure 3(d)). Addition of exendin-4 to 16,7 mmol/l glucose in both groups did not significantly alter MTT reduction. We also tested the response to the sulfonylurea secretagogue tolbutamide. The response to tolbutamide was minimal in control cells but clearly present in cells with Eny2 suppression (Figure 3(e)). Since tolbutamide-induced insulin secretion is not directly dependent on glucose metabolism, this finding suggests additional changes in Eny2 knockdown cells that contribute to the increase in regulated insulin secretion.

### 3.5. Eny2 Suppression Enhances Incretin-Mediated Insulin Secretion Downstream of cAMP

**Figure 4:**
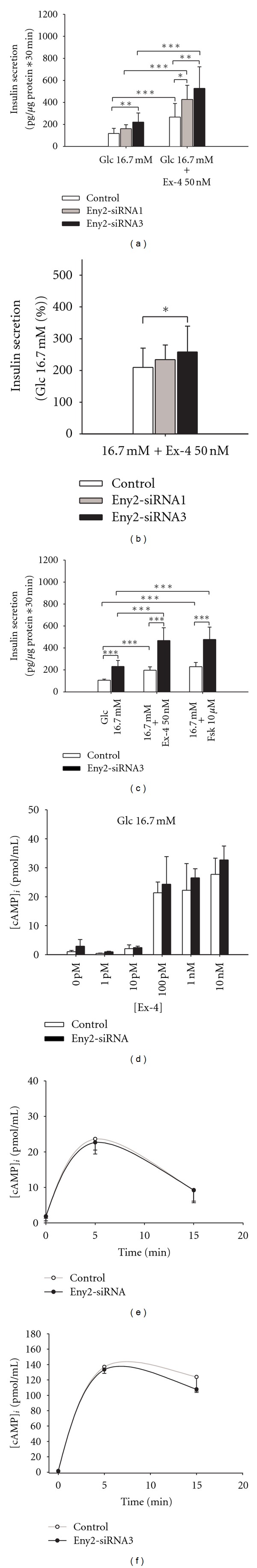
Insulin secretion in response to exendin-4 and forskolin after Eny2 knockdown: levels of cAMP. (a, b) INS-1E cells were stimulated with 50 nmol/l exendin-4 72 hours after transfection with either control or two different Eny2-specific siRNAs. Insulin secretion in response to exendin-4 was augmented by the two specific siRNAs, both in absolute (a) and relative terms (b) (*n* = 5). (c) Insulin secretion after stimulation with 50 nmol/l exendin-4 and 10 *μ*mol/l forskolin, which led to comparable secretory responses. (d) INS-1E cells were stimulated with different concentrations of exendin-4 for 5 minutes. The concentration of cellular cAMP was measured by a competitive ELISA. (e, f) Time course of cellular cAMP levels after stimulation with 50 nmol/l exendin-4 in absence (e) and presence (f) of 100 *μ*mol/l IBMX. In both setups the knockdown of Eny2 did not affect cellular cAMP levels. *P < 0,05, **P < 0,01, ***P < 0,001; one way ANOVA with posttests and unpaired *t*-test.

Incretin signaling via glucagon-like peptide 1 (GLP-1) and gastric insulinotropic peptide (GIP) is a central physiologic regulator of insulin secretion in vivo [15]. We therefore tested whether suppression of Eny2 also affects this pathway. Eny2 siRNA and control transfected cells were stimulated with 50 nmol/l exendin-4 in addition to 16,7 mmol/l glucose. In this experiment suppression of Eny2 increased the exendin-4 induced augmentation of insulin secretion, both in absolute (Figure 4(a)) and in relative terms (Figure 4(b)). A comparable effect was achieved with the adenylyl cyclase activator forskolin (Figure 4(c)). To further evaluate the mechanisms by which Eny2 affects incretin signaling we measured cellular cAMP levels after stimulation with exendin-4. The dose-response curve 5 minutes after stimulation and the time course of cAMP accumulation were not affected by Eny2 knockdown (Figures 4(d)–4(f)). This indicated that Eny2 suppression acts downstream of cAMP.

### 3.6. Eny2 Knockdown Exerts Its Effect on Incretin Signaling Mainly via Modulation of PKA and to a Lesser Extent of EPAC Signaling

**Figure 5:**
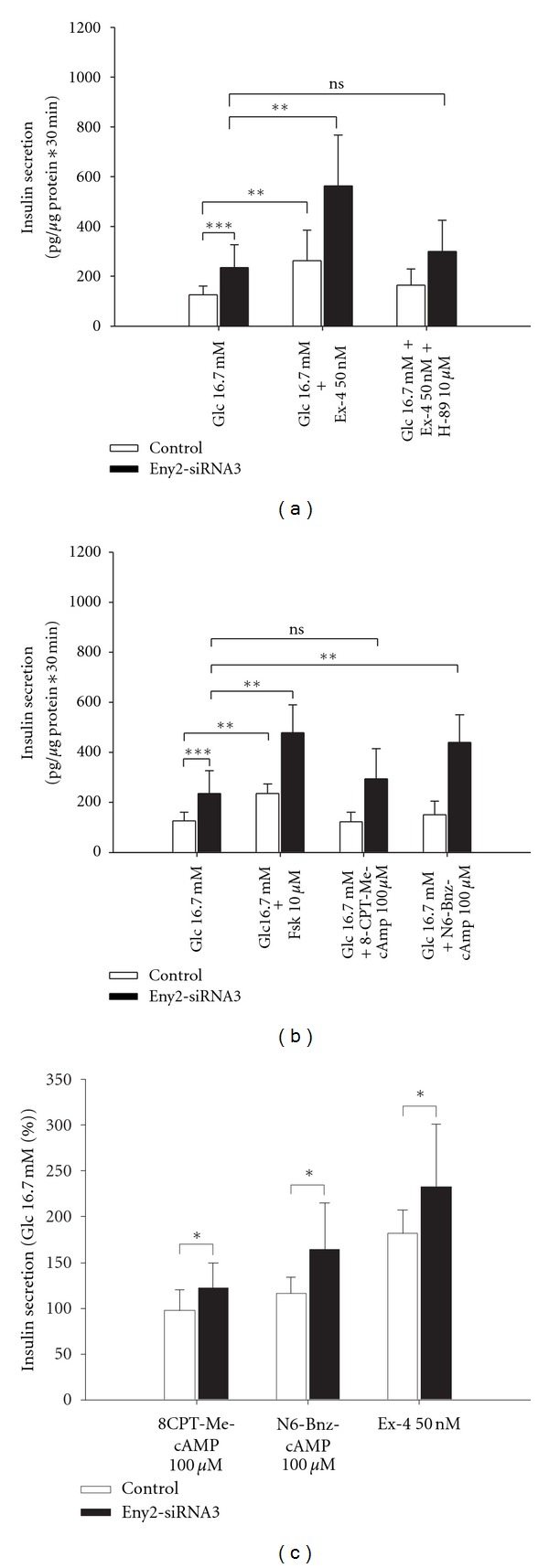
Exendin-4 signaling downstream of cAMP, via PKA and EPAC. (a) Insulin secretion from INS-1E cells treated with 50 nmol/l exendin-4 with and without the PKA inhibitor H-89. Exendin-4 induced insulin secretion from Eny2-knockdown cells was reduced by 80% with PKA inhibition. (b, c) Insulin secretion after stimulation with forskolin, the EPAC-specific activator 8-CPT-Me-cAMP, and the PKA-specific activator N6-Bnz-cAMP; ((b) absolute values; (c) secreted insulin relative to 16.7 mmol/l glucose alone). PKA-specific activation was enhanced in Eny2 knockdown cells, both in absolute insulin values and relative to 16.7 mM glucose alone. The EPAC activator 8-CPT-Me-cAMP was active in cells after Eny2 suppression but not in control cells. This effect was not statistically significant with respect to absolute insulin values but in relative terms (c). *P < 0,05, **P < 0,01, ***P < 0,001; one-way ANOVA with posttests and unpaired *t*-test.

The cAMP signal in beta cells is mainly transmitted by two pathways, the protein kinase A (PKA) and the EPAC pathway [16]. To investigate whether Eny2 suppression specifically affects one of these two pathways we first compared insulin secretion after exendin-4 stimulation with and without the PKA inhibitor H-89. H-89 reduced the effect of exendin-4 stimulation in Eny2 knockdown cells by about 80% (Figure 5(a)). We also tested specific activators of EPAC and PKA, 8-CPT-Me-cAMP, and N6-Bnz-cAMP and compared those to forskolin (Figures 5(b) and 5(c)). In this experiment the EPAC activator induced a small increase of insulin release in Eny2 knockdown but not in control cells. This effect was not statistically significant in terms of absolute insulin values. However, when the relative increase over 16,7 mM glucose alone was analyzed, the effect of the EPAC activator was significantly greater in cells after Eny2 suppression than in control cells (Figure 5(c)). Activation of insulin secretion by the PKA activator N6-Bnz-cAMP was significantly enhanced in Eny2 knockdown compared to control cells, both in absolute insulin values and relative to 16.7 mM glucose alone (Figures 5(b) and 5(c)).

## 4. Discussion

In this study we show that the knockdown of the protein Eny2 results in increased glucose and incretin-stimulated insulin secretion from at baseline poorly responsive INS-1E insulinoma cells. Eny2 is a multifunctional nuclear factor that is involved in the initiation of gene transcription, mRNP formation, and mRNA export from the nucleus [9]. It participates in several different nuclear protein complexes [5–8] and has been described as a coupling factor that helps connecting gene transcription and mRNA transport [9]. It would seem logical that suppressing such a protein disrupts cellular function; however, at least in transient siRNA transfections over 72–96 hours and with no complete suppression of the Eny2 mRNA, this was not the case in our study. Our results were obtained with two Eny2-specific siRNAs with confirmed activity and a scrambled siRNA control was used in all experiments. We therefore believe that off-target effects can reasonably be excluded. The best results were obtained with an siRNA with only partial suppressive activity. This suggests that an ideal level of Eny2 exists for efficient regulated insulin secretion and that poorly functional INS-1E cells possibly accumulated an overabundance of the protein.

We show that Eny2 suppression leads to higher glucose-dependent production of cellular reducing equivalents. This can in part account for the observed increased GSIS [13]. Our results with the glucose-independent secretagogue tolbutamide however suggest additional cellular mechanisms. With respect to incretin signaling our data indicate that Eny2 suppression acts downstream of cAMP, mainly via the PKA and to a smaller extent via the EPAC pathway. However, we have not yet determined the exact molecular mechanism of that effect.

The INS-1E cells in our lab turned out to be significantly less glucose responsive than the parental INS-1E line [11]. Subtle differences in culture conditions or the preferential growth of poorly glucose responsive INS-1E subclones are probably responsible. Whether our results can be replicated in fully functional INS-1E cells needs to be determined in additional studies.

In several aspects our findings are similar to the results of previous studies that compared subclones of Ins-1 cells with different responsiveness to glucose [17]. Pyruvate cycling pathways were found to correlate with GSIS in the different clones [18]. These metabolic pathways result in the accumulation of cytoplasmatic NADPH, which is in good agreement with our measurements of reducing equivalents in the MTT assay [12, 14]. In another report Yang et al. find that enhanced PKA signaling is linked to increased glucose and forskolin-induced insulin secretion in the same Ins-1 sublines [19]. This is also the case after Eny2 knockdown in our INS-1E cells.

Our study has three main limitations: first of all we used an in vitro system and a clonal cell line and therefore can only speculate on the relevance of our findings in vivo. Second, no antibody against mammalian Eny2 is currently available. We therefore had to rely on mRNA measurements to quantify Eny2 expression. Third, a gap of knowledge still remains between the established nuclear functions of Eny2 and the cellular phenotype we observed upon its suppression. Due to the multiple roles of Eny2 this gap cannot be easily closed. Transcriptomics, proteomics, or functional screening with an siRNA library would be reasonable starting points.

## 5. Conclusions

We identified an unexpected link between the nuclear protein Eny2 and insulin secretion from a poorly glucose-responsive INS-1E subline. Suppression of Eny2 results in increased glucose and incretin-induced insulin release in these cells. Whether these findings extend to other experimental conditions or to in vivo physiology needs to be determined in further studies.

## References

[1] Gerich JE (2003). Contributions of insulin-resistance and insulin-secretory defects to the pathogenesis of type 2 diabetes mellitus. *Mayo Clinic Proceedings*.

[2] Kahn SE, Zraika S, Utzschneider KM, Hull RL (2009). The beta cell lesion in type 2 diabetes: there has to be a primary functional abnormality. *Diabetologia*.

[3] Voight BF, Scott LJ, Steinthorsdottir V (2010). Twelve type 2 diabetes susceptibility loci identified through large-scale association analysis. *Nature Genetics*.

[4] Leahy JL, Hirsch IB, Peterson KA, Schneider D (2010). Targeting *β*-cell function early in the course of therapy for type 2 diabetes mellitus. *Journal of Clinical Endocrinology and Metabolism*.

[5] Zhao Y, Lang G, Ito S (2008). A TFTC/STAGA module mediates histone H2A and H2B deubiquitination, coactivates nuclear receptors, and counteracts heterochromatin silencing. *Molecular Cell*.

[6] Jani D, Lutz S, Marshall NJ (2009). Sus1, Cdc31, and the Sac3 CID region form a conserved interaction platform that promotes nuclear pore association and mRNA export. *Molecular Cell*.

[7] Kopytova DV, Orlova AV, Krasnov AN (2010). Multifunctional factor ENY2 is associated with the THO complex and promotes its recruitment onto nascent mRNA. *Genes and Development*.

[8] Kurshakova M, Maksimenko O, Golovnin A (2007). Evolutionarily conserved E(y)2/Sus1 protein is essential for the barrier activity of Su(Hw)-dependent insulators in Drosophila. *Molecular Cell*.

[9] Kopytova DV, Krasnov AN, Orlova AV (2010). Eny2: couple, triple...more?. *Cell Cycle*.

[10] Puff R, Dames P, Weise M, Göke B, Parhofer KG, Lechner A (2010). No non-redundant function of suppressor of cytokine signaling 2 in insulin producing *β*-cells. *Islets*.

[11] Merglen A, Theander S, Rubi B, Chaffard G, Wollheim CB, Maechler P (2004). Glucose sensitivity and metabolism-secretion coupling studied during two-year continuous culture in INS-1E insulinoma cells. *Endocrinology*.

[12] Janjic D, Wollheim CB (1992). Islet cell metabolism is reflected by the MTT (tetrazolium) colorimetric assay. *Diabetologia*.

[13] Jitrapakdee S, Wutthisathapornchai A, Wallace JC, MacDonald MJ (2010). Regulation of insulin secretion: role of mitochondrial signalling. *Diabetologia*.

[14] Segu VBG, Li G, Metz SA (1998). Use of a soluble tetrazolium compound to assay metabolic activation of intact *β* cells. *Metabolism*.

[15] Holst JJ, Gromada J (2004). Role of incretin hormones in the regulation of insulin secretion in diabetic and nondiabetic humans. *American Journal of Physiology—Endocrinology and Metabolism*.

[16] Holz GG (2004). Epac: a new camp-binding protein in support of glucagon-like peptide-1 receptor-mediated signal transduction in the pancreatic *β*-cell. *Diabetes*.

[17] Hohmeier HE, Mulder H, Chen G, Henkel-Rieger R, Prentki M, Newgard CB (2000). Isolation of INS-1-derived cell lines with robust ATP-sensitive K+ channel-dependent and -independent glucose-stimulated insulin secretion. *Diabetes*.

[18] Lu D, Mulder H, Zhao P (2002). 13C NMR isotopomer analysis reveals a connection between pyruvate cycling and glucose-stimulated insulin secretion (GSIS). *Proceedings of the National Academy of Sciences of the United States of America*.

[19] Yang S, Fransson U, Fagerhus L (2004). Enhanced cAMP protein kinase A signaling determines improved insulin secretion in a clonal insulin-producing *β*-cell line (INS-1 832/13). *Molecular Endocrinology*.

